# miR‐134 inhibits non‐small cell lung cancer growth by targeting the epidermal growth factor receptor

**DOI:** 10.1111/jcmm.12889

**Published:** 2016-05-31

**Authors:** Qin Qin, Furong Wei, Jianbo Zhang, Xingwu Wang, Baosheng Li

**Affiliations:** ^1^ Department of Radiation Oncology (Chest Section)Shandong Cancer Hospital and InstituteShandong UniversityJinanChina; ^2^ Department of Radiation Oncology (Chest Section)Shandong Cancer Hospital and InstituteShandong Academy of Medical SciencesJinanChina; ^3^ Institute of Basic MedicineShandong Academy of Medical SciencesSchool of Medicine and Life SciencesUniversity of Jinan‐Shandong Academy of Medical SciencesJinanChina; ^4^ Department of PathologyShandong Cancer Hospital and InstituteShandong UniversityJinanChina; ^5^ Basic Research CenterShandong Cancer Hospital and InstituteShandong UniversityJinanChina

**Keywords:** EGFR, non‐small cell lung cancer, miR‐134, tumour suppressor

## Abstract

The epidermal growth factor receptor (EGFR) is frequently activated in a wide range of solid tumours and represents an important therapeutic target. MicroRNAs (miRNAs) have recently been recognized as a rational and potential modality for anti‐EGFR therapies. However, more EGFR‐targeting miRNAs need to be explored. In this study, we identified a novel EGFR‐targeting miRNA, miRNA‐134 (miR‐134), in non‐small‐cell lung cancer (NSCLC) cell lines. Luciferase assays confirmed that EGFR is a direct target of miR‐134. In addition, the overexpression of miR‐134 inhibited EGFR‐related signaling and suppressed NSCLC cells proliferation by inducing cell cycle arrest and/or apoptosis, suggesting that miR‐134 functions as a tumour suppressor in NSCLC. Further mechanistic investigation including RNAi and rescue experiments suggested that the down‐regulation of EGFR by miR‐134 partially contributes to the antiproliferative role of miR‐134. Last, *in vivo* experiments demonstrated that miR‐134 suppressed tumour growth of A549 xenograft in nude mice. Taken together, our findings suggest that miR‐134 inhibits non‐small cell lung cancer growth by targeting the EGFR.

## Introduction

Lung cancer is the leading cause of cancer‐related deaths worldwide 1, and more than 85% of lung cancers are currently classified as non‐small‐cell lung cancer (NSCLC) 2. The epidermal growth factor receptor (EGFR) belongs to the ERBB family, which is frequently aberrantly activated in a wide range of solid tumours including NSCLC 3. Epidermal growth factor receptor has been recognized as an effective anti‐cancer target for decades 4, 5, and EGFR‐targeted therapies, including monoclonal antibodies (mAbs) and small‐molecule tyrosine kinase inhibitors (TKIs), have been successfully applied in the clinic, showing promising outcome in selected patients 6, 7, 8, 9. However, innate or acquired resistance to these EGFR‐targeted therapies remains a significant challenge, resulting in treatment failure and necessitating more effective targeted strategies 3, 10, 11.

MicroRNAs (miRNAs) are small non‐coding RNAs of approximately 22 nucleotides that post‐transcriptionally regulate gene expression 12, 13. Increasing evidence suggests that miRNAs are involved in many human diseases, particularly cancer. Aberrant miRNA expression profiles are frequently observed in cancers 14, 15, 16, and many miRNAs are implicated in the initiation and progression of cancer and represent potential targets for anti‐cancer treatment 17, 18. MicroRNA‐based therapy has been suggested to be a rational and potential approach for the therapeutic targeting of EGFR 19. To date, a number of miRNAs, such as miR‐7 20, miR‐27b 21 and miR‐133a 22 have been demonstrated to directly target EGFR. However, more anti‐EGFR miRNAs need to be explored, as a single mRNA can be the target of hundreds of miRNAs, and combinations of multiple tumour suppressive miRNAs targeting an individual gene might improve therapeutic efficacy by reducing resistance 17.

In this study, we aimed to identify novel EGFR‐targeting miRNAs and to determine the biological function of miRNAs that are identified in NSCLC both *in vitro* and *in vivo*.

## Materials and methods

### Cell culture

Human NSCLC cell lines (A549, NCI‐H1299, NCI‐H460, NCI‐H520, PC9 and NCI‐H1975) were purchased from the Type Culture Collection of the Chinese Academy of Sciences (Shanghai, China) from 2010 to 2012. However, only two cell lines (A549 and PC9) have been recently tested and authenticated in December 2014 using the short tandem repeat analysis method using Promega PowerPlex1.2 analysis system (Genewiz Inc, Beijing, China). The cells were cultured in RPMI 1640 medium supplemented with 10% foetal bovine serum (FBS), 100 units/ml of penicillin, and 100 μg/ml of streptomycin and incubated in 5% CO_2_ at 37°C. For transfection and subsequent experiments, the cells were pre‐cultured in 10% FBS RPMI 1640 medium, followed by transfection and incubation in 5% FBS‐containing RPMI 1640 medium until the start of the experiments.

### miRNA and siRNA transfection

Cells were cultured to 40–50% confluence and transiently transfected with 50 nM of negative control (NC) miRNA (miR‐NC) or miRNA mimics (miR‐7, miR‐134, miR‐200a, miR‐373) using HiPerFect Transfection Reagent (Qiagen, Germantown, MD, USA) according to the manufacturer's protocols. As for the transfection of miR‐134 inhibitors (anti‐miR‐134) and the corresponding NC (anti‐NC), we used a final concentration of 100 nM. For the RNAi experiments, siRNAs were also transfected using HiperFect Transfection Reagent with a final concentration of 50 nM. MicroRNA mimics and NC were purchased from GenePharma (Shanghai, China), and the siRNAs were purchased from RiboBio (Guangzhou, China).

### Western blotting

Cells were lysed using a cell lysis buffer for Western blotting and IP (Beyotime, Shanghai, China) with protease inhibitor PMSF (Beyotime). Equal amounts of protein were separated by SDS‐PAGE and then transferred onto polyvinylidene difluoride membranes (Millipore, Billerica, MA, USA). The membranes were blocked with 5% nonfat milk in Tris buffered saline (TBS) containing 0.1% Tween 20 and then incubated with the indicated primary antibodies. Primary antibodies against EGFR, phosphorylated EGFR (p‐EGFR; Tyr1068), p‐STAT3 (Tyr705) and GAPDH (Abcam, Cambridge, UK) were used at a dilution of 1:10,000. Primary antibodies against p‐AKT (Ser473; Immunoway, Staffordshire, UK) and p‐ERK1/2 (Thr202/204; Cell Signaling Technology, Shanghai, China) were was used at dilutions of 1:1000 and 1:4000 respectively. Horseradish peroxidase (HRP)‐conjugated secondary antibodies (anti‐mouse IgG and anti‐rabbit IgG) were used to detect the primary antibodies. An ECL chemiluminescence kit (CWBIO, Beijing, China) was used to detect HRP.

### RNA extraction and quantitative real‐time PCR

Total RNA containing miRNAs was isolated from cells using the miRNeasy Mini Kit (Qiagen). For single‐stranded complementary DNA synthesis, 500 ng of total RNA was reverse‐transcribed using PrimeScript^™^ RT reagent Kit with gDNA Eraser (TARAKA, Dalian, China). Real‐time PCR was performed with UltraSYBR Mixture (CWBIO) and LC 480 PCR System (Roche, Shanghai, China). Epidermal growth factor receptor primers were designed as follows: forward, 5′‐GTGGCGGGACATAGTCAGCA‐3′; reverse, 5′‐CCCATTGGGACAGCTTGGA‐3′. GAPDH primers were designed as follows: forward, 5′‐CATGAGAAGTATGACAACAGCCT‐3′; reverse, 5′‐AGTCCTTCCACGATACCAAAGT‐3′. For quantification of miRNA, 500 ng of total RNA was reverse‐transcribed using the miScript II RT kit (Qiagen), and miR‐134 PCR amplification was performed with specific primers and the miScript SYBR Green PCR Kit (Qiagen). U6 small nuclear RNA (RNU6) was used as the endogenous control. The expression levels of EGFR and miR‐134 were normalized to the endogenous controls GAPDH and RNU6, respectively, using the 2^−ΔΔCt^ methods.

### Luciferase reporter assay

The wild‐type 3′UTR of EGFR and its target‐site mutant 3′UTR were amplified by PCR, and the PCR products were cloned into the XhoI/NotI site of the psiCHECK‐2 dual luciferase reporter plasmid (Promega, Madison, WI, USA). These vectors were named psiCHECK‐2‐EGFR‐3′UTR and psiCHECK‐2‐EGFR‐3′UTRm respectively. To perform the luciferase reporter assay, HEK293T cells were plated into 96‐well plates and cotransfected with the reporter vectors and 50 nM of miR‐NC or miR‐134 mimics using Attractene Transfection Reagent (Qiagen). At 48 hrs after transfection, the Firefly and Renilla luciferase activities were measured using a dual‐luciferase reporter system (Promega).

### Cell proliferation assay

The effect of miR‐134 on cell proliferation was evaluated by MTT assay. Briefly, 2 × 10^3^ cells were grown in complete medium in 96‐well plates overnight and then transfected with miR‐NC or miR‐134 mimics at a final concentration of 50 nM. At 0–4 days after transfection, 20 μl of 5 mg/ml MTT (Sigma‐Aldrich, St. Louis, MO, USA) was added to each well. Four hours after incubation with the MTT, the supernatants were discarded, and the formazan precipitates were dissolved in 100 μl of dimethylsulphoxide. The solutions were measured using a microplate reader at 570 nm.

### Assessment of cell death and apoptosis

Cell death and apoptosis were assessed using an Annexin V‐FITC/PI kit (CWBIO) by flow cytometry. At 72 hrs after transfection with miR‐NC or miR‐134 mimics, the cells were harvested and washed with PBS and then stained with Annexin V‐FITC and PI according to the manufacturer's protocol. Cell samples were analysed using a FACscan, and the apoptotic and dead cell fractions were determined.

### Cell cycle analysis

The cell cycle status was assessed by propidium iodide flow cytometry. Briefly, the cells were transfected with miR‐NC or miR‐134 mimic for 72 hrs. Next, the cells were harvested, washed with PBS, and fixed in 70% (v/v) ethanol. Ethanol‐fixed cells were treated with RNaseA and stained with propidium iodide for 30 min. at 4°C. The distribution of cells in different phases of the cell cycle (G0/G1, S and G2/M) was analysed using a FACscan (Becton Dickinson, Franklin Lakes, NJ, USA).

### Generation of A549 and H1299 cells stably expressing EGFR

Lentiviral vectors that express EGFR or empty lentiviral vectors were purchased from GeneChem (Shanghai, China). Cellular infections were performed following the manufacturer's protocol. Cells stably overexpressing EGFR or empty vector were selected by puromycin (2 μg/ml). A549 and H1299 cells that stably expressed empty vector or EGFR were designated as A549‐control, H1299‐control, A549‐EGFR and H1299‐EGFR.

### Functional rescue experiments

Rescue experiments were performed to determine whether EGFR mediates the tumour suppressive effects of miR‐134. A549‐control, A549‐EGFR, H1299‐control and H1299‐EGFR cells were transfected with miR‐NC or miR‐134 mimics, followed by assessment by MTT assays.

### Animal experiments

To establish a lung cancer xenograft model, 2 × 10^6^ A549 cells suspended in 100 μl of phosphate‐buffered saline were injected subcutaneously in the right hindlimbs of BALB/c nude mice (female, 4–5 weeks old, purchased from Beijing HFK Bioscience Co., Ltd, China). After 10 days, when the tumour diameters reached approximately 5–6 mm, the nude mice were randomly divided into two groups (*n* = 4 each). miR‐134 agomir or NC agomir (RiboBio Co., Ltd, Guangzhou, China) was then directly injected into the implanted tumour at a dose of 5 nmol per mouse every 3 days for 15 days. Tumour volume (V) was monitored every 3 days after the first day of agomir injection by measuring the tumour length (L) and width (W) with a vernier caliper and calculated using the formula V = 0.5 × L × W^2^. At 48 hrs after the last injection, the animals were sacrificed, and the tumour tissues were resected. The mice were manipulated and housed according to protocols approved by Shandong Hospital Experimental Animal Care Commission.

### Immunohistochemistry

Tumour tissues were fixed in formalin and imbedded in paraffin. Five‐micron‐thick sections were cut from the embedded tissues and mounted on polylysine‐coated slides. In addition to standard staining with haematoxylin and eosin, the tumour sections were subjected to immunohistochemistry (IHC) staining to detect EGFR, Ki‐67 and cleaved PARP. Briefly, the sections were deparaffinized in xylene, rehydrated in a gradient of alcohol, and treated with 0.3% H_2_O_2_ for 15 min. to quench endogenous peroxidase activity. Following antigen retrieval, the sections were blocked in 10% normal serum with 1% bovine serum albumin in TBS for 2 hrs at room temperature, followed by incubation at 4°C overnight with the indicated primary antibodies (EGFR cst4267, Ki‐67 ab92742, Cleaved PARP ab32064). Negative controls were incubated with NC antibody under the same conditions. Next, the sections were incubated with biotinylated secondary antibody for 1 hr, followed by incubation with conjugated HRP streptavidin for 1 hr. Last, the sections were incubated with diaminobenzidine and counterstained with haematoxylin.

### Statistical analysis

Experiments were performed at least three times. The data were analysed by Student's *t*‐test when comparing two groups and by one‐way anova followed by Bonferroni post test when comparing more than two groups. *P* < 0.05 were considered statistically significant.

## Results

### miR‐134 down‐regulates EGFR expression in NSCLC cell lines

To identify novel miRNAs that regulate EGFR expression, we used a computational algorithm (microrna.org) to select potential miRNAs for assessment. Among the predicted conserved miRNAs with favourable mirSVR scores, we focused on those miRNAs that function as tumour suppressors but that have not been identified to regulate EGFR. Three miRNAs (miR‐134, miR‐200a and miR‐373) were selected for experimental validation, with the well‐characterized EGFR repressor miR‐7 as a positive control.

**Figure 1:**
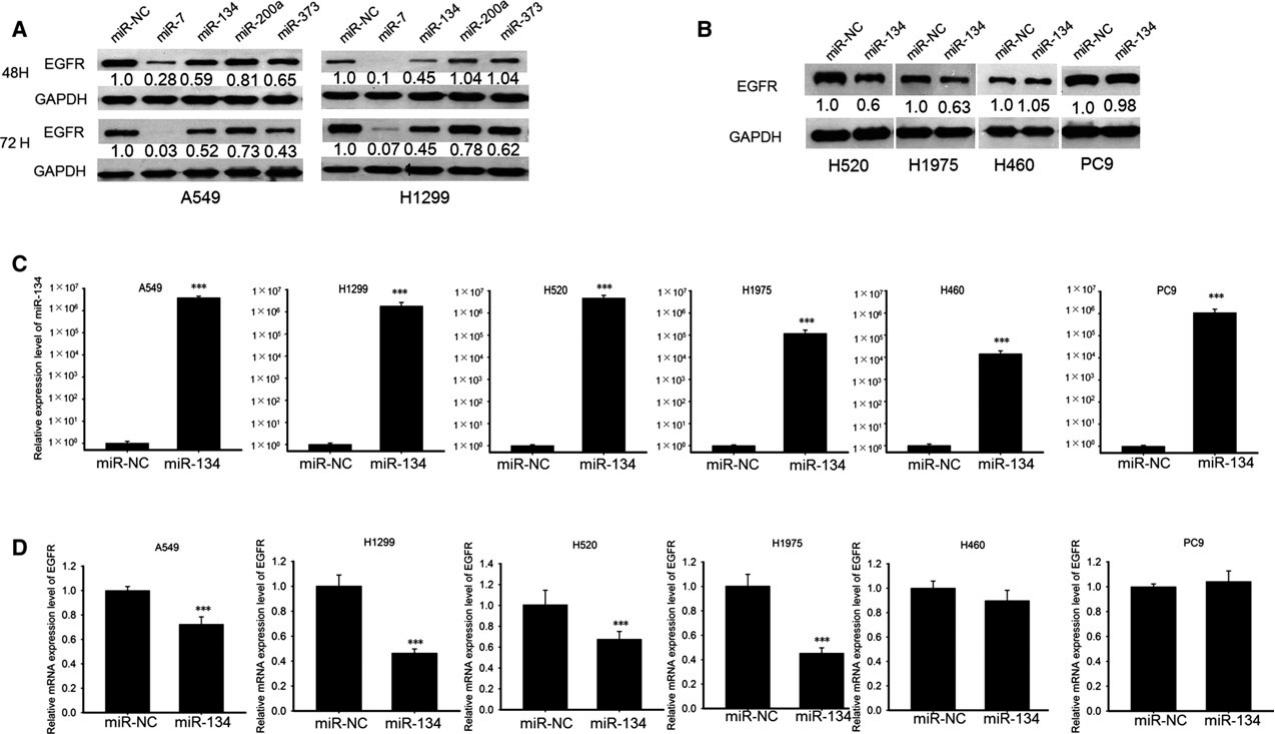
miR‐134 down‐regulates EGFR expression in NSCLC cell lines. (**A**) EGFR protein levels in NSCLC cell lines (A549 and H1299) at 48 and 72 hrs after transfection with miR‐NC, miR‐7, miR‐134, miR‐200a and miR‐373 mimics. (**B**) EGFR protein levels in NSCLC cell lines (H520, H1975, H460 and PC9) at 72 hrs after transfection of miR‐NC or miR‐134 mimics. (**C**) miR‐134 expression levels were significantly up‐regulated after transfection with 50 nM of miR‐134 mimics. (**D**) EGFR mRNA levels were decreased at 48 hrs after transfection with miR‐134 in A549, H1299, H520 and H1975 cells. Experiments represent three replicates, and the data are shown as the mean values ± S.D. ***P < 0.001.

For the initial assessment, we transfected two NSCLC cell lines (A549 and H1299) with miRNA mimics. Next, western blotting was performed to investigate EGFR expression at 48 and 72 hrs after transfection. As shown in Figure 1A, miR‐7 down‐regulated EGFR expression significantly at 48 and 72 hrs after transfection. Among the three tested miRNAs, miR‐134 exerted the most significant inhibitory effect on EGFR expression in both cell lines at 48 and 72 hrs after transfection. Therefore, we chose miR‐134 for further investigation by western blotting at 72 hrs after transfection (as the down‐regulation of EGFR at 72 was more significant than at 48 hrs after transfection.

To further validate the inhibitory effect of miR‐134 on EGFR expression in lung cancer cells, we transfected four additional NSCLC cell lines, H460, H520, H1975 and PC9, with miR‐134 mimics. As shown in Figure 1B, miR‐134 inhibited EGFR expression in H520 and H1975 but not in H460 and PC9 cells at 72 hrs after transfection. We also performed qRT‐PCR to detect alterations in EGFR mRNA levels at 48 hrs after transfection. As shown in Figure 1C and 1D, transfected cells exhibited significantly increased levels of miR‐134; transfection of miR‐134 inhibited EGFR mRNA levels in A549, H1299, H520 and H1975 but not H460 and PC9 cells.

On the basis of these results showing that miR‐134 inhibited EGFR expression in A549, H1299, H520 and H1975 cells, we further transfected these cells with anti‐miR‐134 to test whether inhibition of miR‐134 would affect the expression of EGFR. As shown in Figure S1, transfection of anti‐miR‐134 up‐regulated EGFR protein levels significantly in those cell lines, whereas no apparent up‐regulation of EGFR mRNA expression was observed.

Taken together, the results suggest that miR‐134 down‐regulates EGFR expression in four of the six‐tested NSCLC cell lines.

### EGFR is a direct target of miR‐134

**Figure 2:**
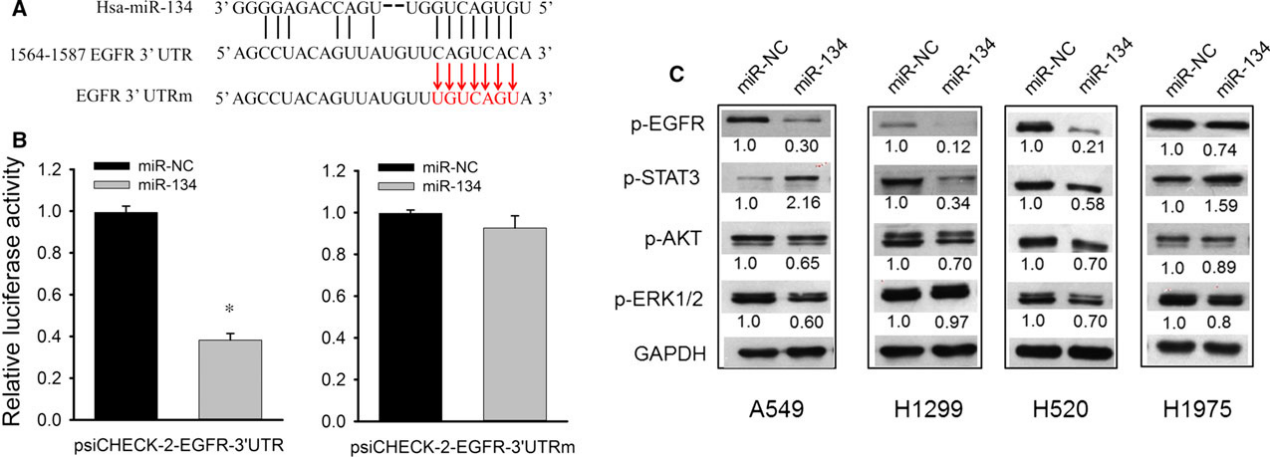
miR‐134 targets EGFR directly and regulates EGFR‐associated signaling in NSCLC cell lines. (**A**) Predicted miR‐134 target site within the EGFR 3′UTR. (**B**) Luciferase reporter assays were performed in HEK293T cells by cotransfection of miR‐NC or miR‐134 mimics with reporter vectors psiCHECK‐2‐EGFR‐3′UTR or psiCHECK‐2‐EGFR‐3′UTRm. Experiments were represent three replicates, and the data are shown as the mean values ± S.D. *P < 0.05. (**C**) p‐STAT3, p‐AKT and p‐ERK1/2 that represent the major signaling pathways downstream of EGFR were detected by western blotting in A549, H1299, H520 and H1975 cells.

To determine whether down‐regulated EGFR expression levels were due to the direct targeting of miR‐134 to the EGFR 3′UTR, we constructed luciferase reporter vectors containing wild‐type (psiCHECK‐2‐EGFR‐3′UTR) and target‐site mutant (psiCHECK‐2‐EGFR‐3′UTRm) 3′UTR of EGFR to assess the direct targeting of the EGFR 3′UTR by miR‐134 in HEK293T cells (Fig. 2A). Compared with miR‐NC, cotransfection of miR‐134 and psiCHECK‐2‐EGFR‐3′UTR showed significantly decreased luciferase activity, whereas cotransfection of miR‐134 and psiCHECK‐2‐EGFR‐3′UTRm did not result in significant reduction in luciferase activity (Fig. 2B). These results indicate that EGFR is a direct target of miR‐134.

### miR‐134 suppresses EGFR‐associated signaling in NSCLC cell lines

On the basis of the finding that EGFR is a direct target of miR‐134 and that the overexpression of miR‐134 can inhibit EGFR expression in NSCLC cells, we next investigated whether miR‐134 could suppress EGFR‐associated signaling by down‐regulating EGFR expression. As shown in Figure 2C, although overexpression of miR‐134 resulted in consistent down‐regulation of p‐EGFR in A549, H1299, H520 and H1975 cells, down‐regulation of p‐STAT3, p‐Akt and p‐ERK1/2 were not as concordant as predicted. A549 cells exhibited decreased p‐Akt and p‐ERK1/2 but increased p‐STAT3; H1299 cells exhibited decreased p‐STAT3 and p‐Akt but had no significant impact on p‐ERK1/2; H520 cells exhibited decreased p‐STAT3, p‐Akt and p‐ERK1/2; H1975 cells exhibited decreased p‐ERK1/2 but increased p‐STAT3, whereas no significant changes were detected in p‐Akt.

Collectively, the results suggest that miR‐134 can down‐regulate p‐EGFR in NSCLC cell lines and suppress specific EGFR‐associated signaling in a cellular context‐dependent way.

### miR‐134 inhibits cancer cell proliferation by inducing cell apoptosis and/or cell cycle arrest in G1/G0

**Figure 3:**
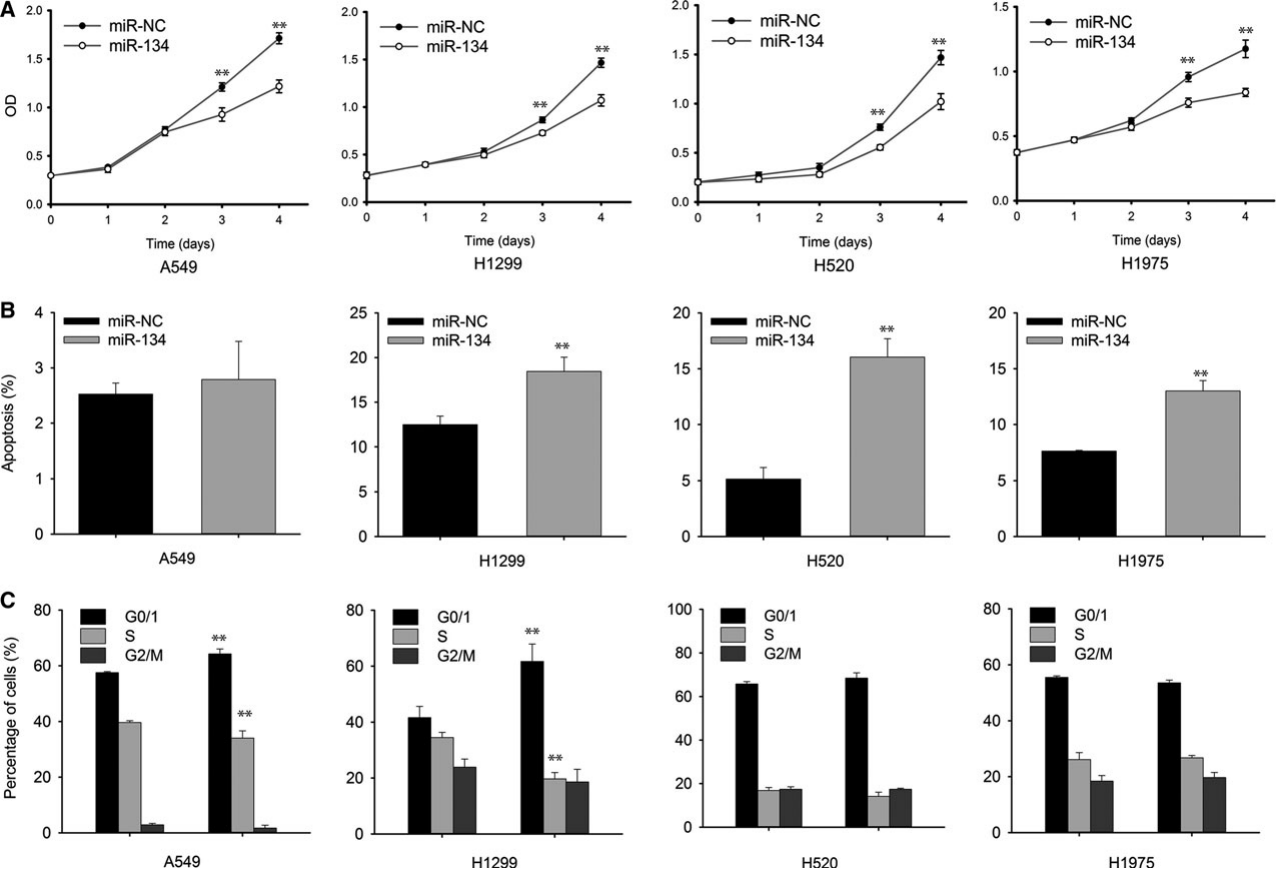
miR‐134 inhibits cell proliferation in NSCLC. (**A**) MTT assays were performed to evaluate cell proliferation at 0–4 days after transfection with miR‐NC or miR‐134 mimics in NSCLC cells (A549, H1299, H520 and H1975). (**B**) Analysis of cell death and apoptosis in NSCLC cells (A549, H1299, H520 and H1975) at 72 hrs after transfection with miR‐NC or miR‐134 mimics. (**C**) Cell cycle analysis of NSCLC cells at 72 hrs after transfection with miR‐NC or miR‐134 mimics. **P < 0.01.

Because EGFR signaling is an important pro‐survival factor in cancer cells, we have been suggested that by down‐regulating EGFR expression, miR‐134 might play an inhibitory role in cancer cell proliferation. To investigate this hypothesis, we transfected the aforementioned 4 NSCLC cell lines (A549, H1299, H520, H1975), in which EGFR signaling was suppressed after miR‐134 transfection, with miR‐134 mimics. As shown in Figure 3A, transfection of miR‐134 significantly inhibited the proliferation of all the assessed cancer cells.

To further investigate, the mechanism underlying the action of miR‐134 on cancer cell growth inhibition, we analysed the effect of miR‐134 on the cell cycle progression and apoptosis. As shown in Figure 3B and C, transfection of miR‐134 in A549 and H1299 cells significantly increased the proportion of cells in G1/G0 phase and significantly decreased the proportion of cells in S phase, whereas no significant changes were detected in the proportion of cells in G2/M phase. However, transfection of H520 and H1975 with miR‐134 did not result in cell cycle arrest. With regard to apoptosis, miR‐134 overexpression significantly increased apoptosis in H1299, H520 and H1975 cells, whereas no significant change was detected in A549 cells. Together, the results indicate that miR‐134 might inhibit proliferation of lung cancer cells by inducing cell apoptosis and/or cell cycle arrest.

### EGFR mediates the effects of miR‐134 on NSCLC proliferation

**Figure 4:**
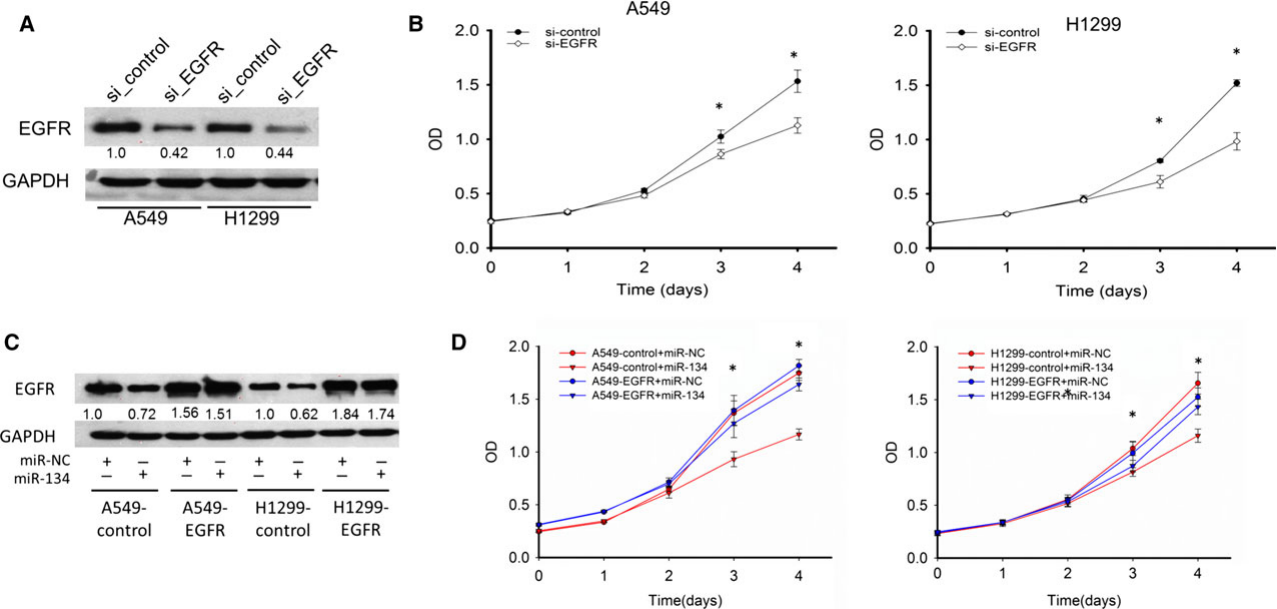
EGFR mediates the effects of miR‐134 on NSCLC cell proliferation. (**A**) Western blotting was performed to confirm the knockdown of EGFR expression in A549 and H1299 after transfection with si_EGFR. (**B**) MTT assays were performed to evaluate cell proliferation at 0–4 days after transfection with si_control or si_EGFR in A549 and H1299, showing that knockdown of EGFR inhibits cell proliferation. (**C**) Western blotting was performed to detect EGFR expression in A549‐control, A549‐EGFR, H1299‐control and H1299‐EGFR cells after transfection with miR‐NC or miR‐134. (**D**) MTT assays were performed to confirm that EGFR overexpression could rescue the suppressive effect of miR‐134 on cell proliferation. The results are shown as the mean values ± S.D. *P < 0.05.

To determine whether miR‐134 exerts its tumour suppressive functions through down‐regulation of EGFR, we first performed RNAi experiments to determine whether EGFR knockdown would mimic the phenotype of miR‐134 overexpression (Fig. 4A). As expected, EGFR knockdown in A549 and H1299 cells significantly suppressed cell proliferation (Fig. 4B).

Next, we performed rescue experiments. We constructed EGFR‐overexpressing A549 and H1299 cells using lentiviral vector (A549‐EGFR and H1299‐EGFR), in which the 3′UTR of EGFR was missing. EGFR overexpression was confirmed by western blotting. Although miR‐134 inhibited EGFR expression in A549 and H1299 cells transfected with lentiviral‐vector control (A549‐control and H1299‐control), no significant down‐regulation of EGFR was detected in A549‐EGFR and H1299‐EGFR cells (Fig. 4C). miR‐134 transfection significantly reduced cell proliferation in A549‐control and H1299‐control but not in A549‐EGFR and H1299‐EGFR (Fig. 4D).

Collectively, EGFR knockdown exhibited a similar phenotype as miR‐134 overexpression, and rescue experiments confirmed that the tumour suppressive function of miR‐134 is mediated partly by down‐regulating EGFR.

### miR‐134 suppresses tumour growth of A549 xenograft in nude mice

**Figure 5:**
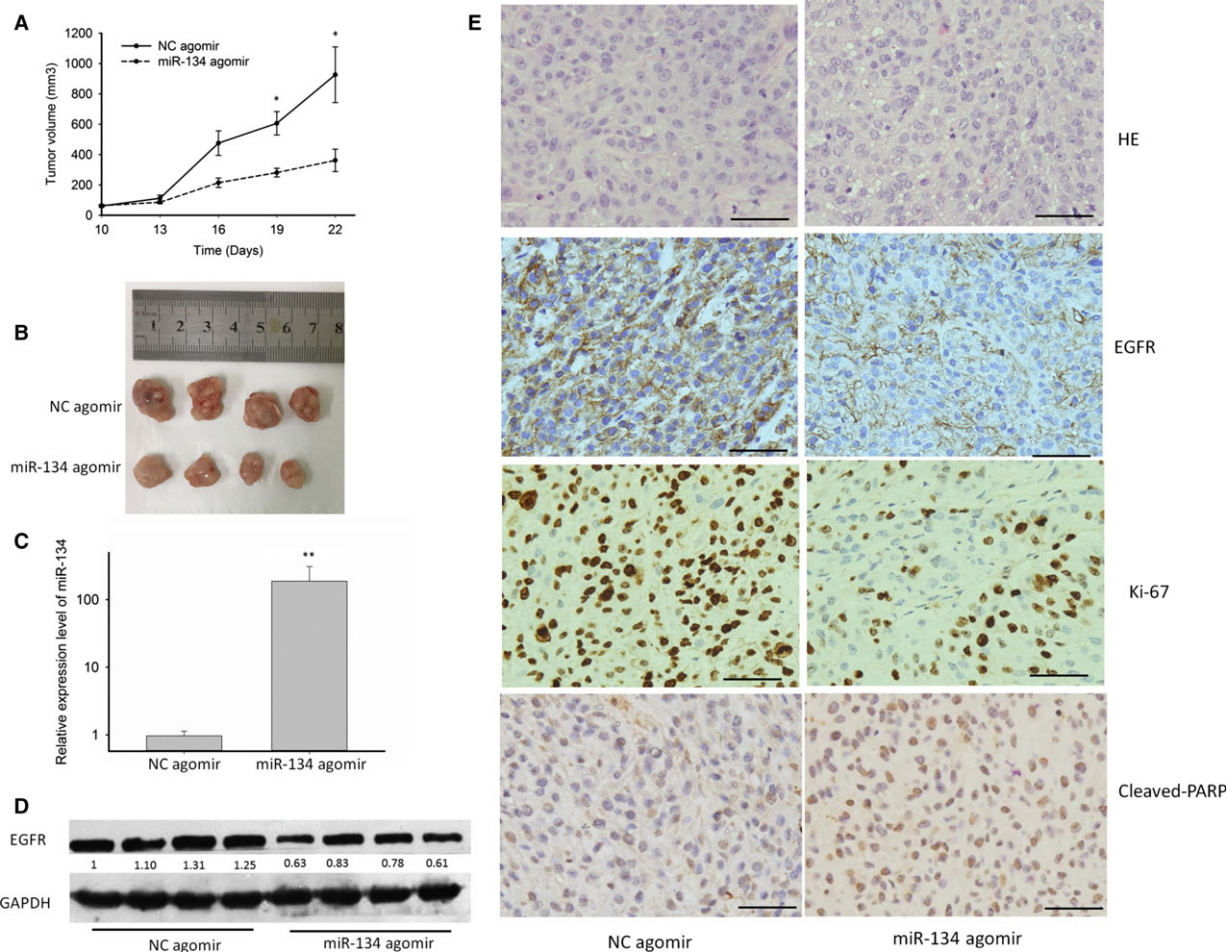
miR‐134 suppresses tumour growth of A549 xenograft in nude mice. (**A**) Tumour growth curves presented by tumour volume indicate that miR‐134 inhibits A549 xenograft growth. (**B**) Tumour images of A549 xenografts injected with NC agomir and miR‐134 agomir. (**C**) miR‐134 expression levels were up‐regulated in xenografts injected with miR‐134 agomir compared with those injected with NC agomir. (**D**) Western blotting shows decreased EGFR expression in xenografts injected with miR‐134 agomir compared with those injected with NC agomir. (**E**) Haematoxylin and eosin and IHC staining of xenograft sections. EGFR expression levels were reduced in xenografts injected with miR‐134; the proliferation marker Ki‐67 was decreased, and the apoptotic marker cleaved PARP was increased in xenografts injected with miR‐134. *P < 0.05, **P < 0.01. Scale bars indicate 50 μm.

On the basis of the tumour suppressive roles of miR‐134 *in vitro*, we continued to investigate the roles of miR‐134 *in vivo* to assess the therapeutic potential of miR‐134. As shown in Figure 5A and B, intratumoural injection with the miR‐134 agomir significantly inhibited the growth of A549 xenografts, compared with the miR‐NC agomir (NC agomir).

To elucidate the underlying mechanisms of miR‐134‐mediated tumour suppression in A549 xenografts, we analysed the expression of miR‐134, EGFR, Ki67 and cleaved PARP in resected A549 xenografts. Although RT‐qPCR confirmed the elevated expression of miR‐134 in A549 xenografts injected with the miR‐134 agomir (Fig. 5C), western blotting and IHC revealed the decreased the expression of EGFR (Fig. 5D and E). Consistent with the suppressed tumour growth in A549 xenografts injected with the miR‐134 agomir, the expression of Ki‐67 (a cell proliferation marker) was decreased, whereas the expression of cleaved PARP (marker of apoptosis) was increased (Fig. 5E). Taken together, these data indicate that miR‐134 could inhibit tumour growth *in vivo*, which might be attributed to attenuated cell proliferation and increased apoptosis.

## Discussion

Epidermal growth factor receptor has long been proposed as an attractive and promising target for anti‐cancer treatment 4. In addition to classical mAbs and TKIs for EGFR‐targeted therapies, recent EGFR‐miRNAs regulation network studies highlight the possibility that miRNA‐based therapy could be utilized to target EGFR 19. Identifying miRNAs that inhibit EGFR expression is crucial for effective EGFR targeting. Therefore, we used a computational algorithm to select potential miRNAs and identified miR‐134 as an EGFR‐targeting miRNA. During the preparation of this manuscript, one of our tested miRNAs, miR‐200a, was also confirmed to target EGFR 23.

miR‐134 was first identified as a brain‐specific miRNA that is involved in synapse development 24. Subsequent studies suggested that miR‐134 also plays an essential role in stem cell differentiation 25, 26, 27. Recently, studies have revealed the important roles of miR‐134 in cancer 28, 29, 30, 31, 32, 33, 34, 35, 36, 37. miR‐134 was demonstrated to regulate cell proliferation 29, 30, 33, 35, 36, migration 30, 31, 32, 35, invasion 30, 31, 32 and epithelial‐mesenchymal transition 28, 34, 37 in a number of cancers. However, its roles in NSCLC remain controversial 28, 34, 35. In this study, the overexpression of miR‐134 in NSCLC cells *in vitro* resulted in decreased proliferation and migration as well as invasion, and *in vivo* experiments employing intratumoural injections with miR‐134 agomir inhibited A549 xenograft growth, suggesting that miR‐134 plays a tumour suppressor role in NSCLC.

Epidermal growth factor receptor signaling comprises three major signal transduction pathways, RAS/RAF/MEK/ERK, PI3k/AKT and STAT3‐dependent signaling 4, 11. To determine whether EGFR down‐regulation by miR‐134 can result in weakened EGFR signaling, we examined the activation of p‐EGFR, p‐ERK1/2, p‐AKT and p‐STAT3 in four NSCLC cell lines (A549, H1299, H520 and H1975) after miR‐134 overexpression. As foregoing presented, although p‐EGFR exhibited consistent suppression, inhibition of p‐ERK1/2, p‐AKT and p‐STAT3 was only observed in H520 cells, whereas other cells exhibited decreased activation in one or two of the three investigated signaling pathways. Intriguingly, A549 and H1975 cells exhibited increased p‐STAT3, which primarily mediates pro‐survival and proliferative signaling in cancer cells. Such phenomena cannot be simply attributed to down‐regulated EGFR, implying that other targets of miR‐134 in these cells might be involved in the regulation of STAT3. More comprehensive investigations are required to uncover underlying mechanisms.

Despite not all the investigated EGFR signaling showed concordant down‐regulation in NSCLC cells, at least one EGFR signaling inhibition was observed in this study. Whether down‐regulated EGFR signaling contributes to proliferative suppression was an open question. Therefore, we performed cellular proliferation assays to determine whether miR‐134 overexpression in NSCLC would inhibit cancer cell growth and verified that miR‐134 promotes apoptosis and/or induces cell cycle arrest, inhibiting cancer cell proliferation. To further investigate the role of EGFR in miR‐134‐mediated tumour suppression, we conducted RNAi and functional rescue experiments, which confirmed that down‐regulation of EGFR by miR‐134 partially contributes to the proliferation suppressive role of miR‐134.

As miR‐134 inhibits NSCLC cells proliferation *in vitro*, we further explored the potential of using miR‐134 for tumour suppression *in vivo*. Our xenograft data suggest that intratumoural injection of miR‐134 agomir decreased the expression of EGFR and suppressed tumour growth by inhibiting proliferation and inducing apoptosis of tumour cells, suggesting that miR‐134 could be a potential strategy for EGFR‐targeted therapy.

Although miR‐7 is a well‐characterized miRNA that targets EGFR signaling both directly and indirectly, its role in tumourigenesis is still controversial. Although some studies indicate that miR‐7 inhibits tumour growth by suppressing EGFR signaling (by inhibiting EGFR and Raf1 directly and suppressing ERK1/2, AKT and STAT3 indirectly) 20, 38, 39, other studies have also indicated that miR‐7 is involved in EGFR‐related lung tumourigenesis 40, suggesting that miR‐7 might function in a cell‐type‐specific way. In our experiments, miR‐7 inhibits EGFR expression as well as EGFR signaling (data not shown). Although miR‐7 exerts a more profound suppression of EGFR signaling, the tumour suppressive effects of miR‐7 were not significantly greater than those of miR‐134 (data not shown).

Although we have demonstrated the tumour suppressive role of miR‐134 in NSCLC *via* targeting EGFR, our study does have specific limitations. First, miR‐134 did not down‐regulate EGFR expression in H460 and PC9 cells, and the underlying mechanisms in that regard were not investigated. One possible explanation is that the target sites of miR‐134 within the EGFR 3′UTR might be mutated in these cells, suggesting that miRNA‐based therapy should not be administered indiscriminately. Second, we could not demonstrate the clinical significance of miR‐134, as we do not have tumour samples. However, one study investigating the prognosis of miRNAs in NSCLC provided evidence for a tumour suppressive role of miR‐134, suggesting that high miR‐134 levels correlate with favourable prognosis in lung squamous cell carcinoma (supplementary material of reference 41) 41. Further studies of the clinical significance of miR‐134 are needed.

In conclusion, we have demonstrated that miR‐134 can down‐regulate EGFR expression in NSCLC cell lines and that overexpression of miR‐134 can inhibit EGFR‐related signaling and suppress NSCLC cells proliferation both *in vitro* and *in vivo*. miR‐134 inhibits NSCLC growth by targeting EGFR.

## Conflict of interest

The authors declare that they have no conflict of interest.

## Supporting information


**Figure S1** The effect of miR‐134 inhibition on EGFR expression. (A) Western blotting showed that anti‐miR‐134 up‐regulated EGFR protein expression in NSCLC cell lines. (B) qRT‐PCR showed no EGFR mRNA expression change after transfection with anti‐miR‐134 in NSCLC cell lines.
