# Supplementary figures and images for: miR‐134 inhibits non‐small cell lung cancer growth by targeting the epidermal growth factor receptor
Source: J Cell Mol Med. 2016 May 31;20(10):1974–83. doi: 10.1111/jcmm.12889 (PMC4891324; doi:10.1111/jcmm.12889)

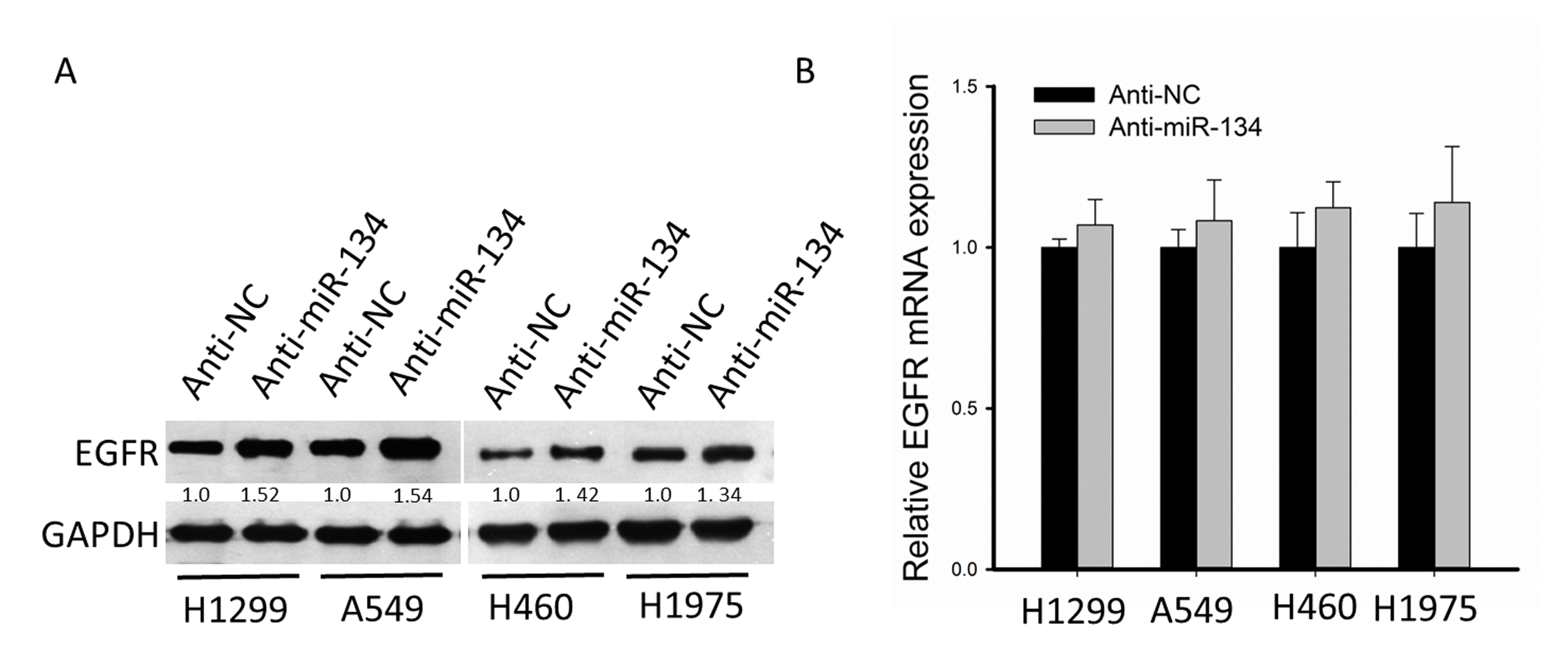

Supplement: Supplementary file 1 — Figure S1 The effect of miR‐134 inhibition on EGFR expression. (A) Western blotting showed that anti‐miR‐134 up‐regulated EGFR protein expression in NSCLC cell lines. (B) qRT‐PCR showed no EGFR mRNA expression change after transfection with anti‐miR‐134 in NSCLC cell lines. [file JCMM-20-1974-s001.tif]
